# Consequences of school closure on access to education: Lessons from the 2013–2016 Ebola pandemic

**DOI:** 10.1007/s11159-021-09900-2

**Published:** 2021-04-26

**Authors:** William C. Smith

**Affiliations:** Moray House School of Education and Sport, University of Edinburgh, Edinburgh, UK

**Keywords:** school closure, health crisis, dropout, Ebola, West Africa, COVID-19, differences-in-differences

## Abstract

The COVID-19 pandemic has seen an unprecedented shutdown of society. Among the various safety measures taken, much attention has been given to school closure as a non-pharmaceutical mitigation tool to curb the spread of the disease through ensuring “social” (physical) distancing. Nearly 1.725 billion children in over 95% of countries worldwide have been affected by school closures implemented in April 2020 as the virus continued to spread. In the field of education, policymakers’ attention has been directed at keeping students on board through remote learning and addressing the immediate needs of schools upon reopening. The study presented in this article focuses on who remains absent after schools resume. Using publicly available survey data from the USAID Demographic Health Surveys Program and the UNICEF Multiple Indicator Cluster Survey from before and after the 2013–2016 Ebola pandemic in Guinea and Sierra Leone in West Africa, the author examined changes in school enrolment and dropout patterns, with targeted consideration given to traditionally marginalised groups. At the time, schools closed for between seven to nine months in the two countries; this length and intensity makes this Ebola pandemic the only health crisis in the recent past to come close to the pandemic-related school closures experienced in 2020. The author’s findings suggest that post-Ebola, youth in the poorest households saw the largest increase in school dropout. Exceeding expected pre-Ebola dropout rates, an additional 17,400 of the poorest secondary-age youth were out of school. This evidence is important for minimising the likely post-COVID-19 expansion in inequality. The author’s findings point to the need for sustainable planning that looks beyond the reopening of educational institutions to include comprehensive financial support packages for groups most likely to be affected.

## Résumé

Conséquences de la fermeture des écoles sur l’accès à l’éducation : les enseignements de la pandémie d’Ebola de 2013 à 2016 **–** La pandémie de COVID-19 a infligé un arrêt sans précédent à la société. Parmi les diverses mesures de sécurité qui ont été prises, une attention particulière à été accordée à la fermeture des écoles devenue un outil non pharmaceutique pour freiner la propagation de la maladie par le biais d’une distanciation « sociale » (physique) ainsi garantie. Près de 1,725 milliard d’enfants dans plus de 95 % des pays du monde entier ont été concernés par les fermetures d’établissements scolaires entrées en vigueur en avril 2020, alors que le virus continuait de se propager. Dans le domaine de l’éducation, les politiques se sont appliquées à permettre aux écoliers de continuer à étudier au moyen de l’apprentissage à distance et à répondre aux besoins immédiats des écoles lors de leur réouverture. L’étude présentée dans cet article est essentiellement consacrée à ceux qui restent absents une fois que l’école a repris. S’appuyant sur des données accessibles au public du Programme d’enquêtes démographiques et sanitaires de l’USAID (l’Agence des États-Unis pour le développement international) et l’Enquête par grappes à indicateurs multiples de l’UNICEF avant et après la pandémie d’Ebola de 2013–2016 en Guinée et en Sierra Leone, en Afrique de l’Ouest, l’auteur s’est penché sur les changements concernant les inscriptions dans les établissements scolaires et les schémas d’abandon de la scolarité en portant une attention particulière aux groupes traditionnellement marginalisés. À cette époque-là, les écoles ont fermé leurs portes pendant sept à neuf mois dans les deux pays. La durée et l’intensité de ces fermetures ont fait de la pandémie d’Ebola la seule crise sanitaire du passé récent à se rapprocher en la matière de celle de COVID-19 en 2020. Les conclusions de l’auteur indiquent qu’après la pandémie d’Ebola, la plus forte hausse des abandons scolaires a été enregistrée chez jeunes des foyers les plus démunis. Dépassant les taux d’abandon d’avant Ebola auxquels on s’attendait, 17 000 écoliers supplémentaires faisant partie des plus pauvres au niveau de l’enseignement secondaire ont abandonné l’école, une preuve importante pour œuvrer à réduire au minimum l’augmentation probable des inégalités après la pandémie de COVID-19. Les conclusions de l’auteur montrent qu’il est nécessaire de planifier durablement en se projetant au-delà de la réouverture des écoles afin d’inclure de vastes aides financières pour les groupes qui seront probablement touchés.

## Introduction

The COVID-19 pandemic is unlike anything the world has seen over the past century.^1^ As of 18 June 2020, nearly 8.5 million individuals have been infected, and almost half a million people have died (Worldometer 2020). In response, and despite resulting economic hardship, governments around the world have shut down society to stem the spread of the disease through “social” (physical) distancing. School closures have been nearly universally used as a mitigation tool. While a recent review found limited research and a marginal association between school closures and disease transmission (Viner et al. 2020), past studies exploring influenza outbreaks have highlighted the potential effectiveness of school closures in slowing down transmission (Jackson et al. 2013), and revealed that affected countries have routinely relied on this measure (UK DOH 2014). At the peak of COVID-19-related school closures in April 2020, over 95% of countries worldwide had fully or partially closed schools, affecting an unprecedented 1.725 billion children around the globe (UNESCO 2020).

At the time of writing,^2^ schools are slowly starting to reopen in some countries, with mixed success. For example, concerns over safety have led England to halt plans to further open primary schools (Coughlan 2020), and in South Korea and Israel, some schools were closed once more after infection rates flared (Gaudiano and Goldberg 2020). Around the world, governments, teachers’ unions and universities are planning for educational institutions to reopen so that face-to-face learning can be resumed. The potential negative consequences of having youth out of school, and the complex challenges of opening facilities during an ongoing pandemic, have focused attention on short-term response and recovery – including ensuring schools and campuses are “COVID-19 secure”, and helping those who return to school make up for lost time in their studies. But what about children and youth who remain absent? In the study I present in this article, I explored the impact of school closures during the 2013–2016 Ebola pandemic^3^ in Guinea and Sierra Leone in West Africa on school enrolment patterns. Drawing on household survey data from directly before and after the pandemic, I used descriptive and inferential statistics to explore: (1) if, and to what extent, school disruptions during the Ebola pandemic in Guinea and Sierra Leone changed the expected pattern for enrolment among school-age youth, and (2) if, and to what extent, alterations disproportionately affected marginalised groups.

The results from this study show a post-pandemic surge in school dropouts concentrated in secondary-age youth from the poorest households in Guinea and Sierra Leone. This effect resulted in an additional 17,410 more youth dropping out of school across the two countries than would have been expected in pre-outbreak times. These findings have implications for education planners as they prepare for recovery from the COVID-19 crisis. By highlighting those students at greatest risk of not returning, this study encourages education systems to adopt a sustainable, targeted approach, using ambitious but realistic timelines as they attempt to restart and rebound.

The next sections provide a review of school disruptions and their relationship with educational outcomes, followed by a look at school closures and past large-scale health crises. The 2013–2016 Ebola pandemic is then introduced, along with the data and methods I used in my analysis. Following a presentation of the results, a discussion section contrasts key characteristics of the Ebola pandemic and the COVID-19 pandemic. In the conclusion, I sum up some preliminary lessons which could prove useful for education planning purposes.

## School disruptions and educational outcomes

School disruptions include both planned and unplanned stoppage of education through the closure of facilities. Common reasons for school disruptions include outbreaks of infectious disease (Cauchemez et al. 2014), natural disasters (Andrabi et al. 2020; Bangkok ADPC 2008; Esnard et al. 2018) and inclement weather (Stuart et al. 2013). In a study of unplanned school closures in the United States (US) between 2011 and 2013, 79% of closures were due to inclement weather, 14% to natural disasters and 4% to infrastructure issues. Only 4% of closures lasted more than four days (Wong et al. 2014).

To see a relationship between school disruptions and educational outcomes, a disruption must be of substantial length. For this reason, most research has examined disruptions due to disasters, school holidays or education transitions (e.g. moving between grades or years). Learning loss over school holidays has been commonly reported (Slade et al. 2017), and has been found to disproportionately affect children in low-income families (Quinn et al. 2016). For example, children who are out of school for the summer holidays in Latin America lose nearly three months of prior learning (Busso and Munoz 2020). In Malawi, Timothy Slade and colleagues (2017) reported a 0.38 standard deviation decrease in reading scores during the three-month transition from Grade 1 to Grade 2. During a similar transition in Ghana, foundational numeracy test differences represented a 66% loss in learning gains, with a complete elimination of learning gains for those without books or reading materials at home (Sabates and Carter 2020).

Lower achievement is also more common among students who have experienced natural disasters. Comparing test scores of students in Thailand who were affected and not affected by flooding, Kawin Thamtanajit (2020) reported a 0.03 to 0.11 standard deviation decline, depending on grade and subject. Research suggests that effects are greater for economically disadvantaged students (Lai et al. 2018) and can persist over time (Andrabi et al. 2020). For instance, four years after the 2005 Pakistan earthquake, family living standards had recovered, but those whose schools had been closed for an average of 80 days experienced learning loss that put them one and a half to two years behind their peers (Andrabi et al. 2020).

Compared to student achievement, enrolment patterns remain understudied. Some researchers have suggested that those living in higher-risk areas for natural disasters are more prone to drop out of education (Bangkok ADPC 2008); however, research relating specifically to health crises and student re-enrolment is relatively rare (see Carvalho et al. 2020 for a review). A study on post-outbreak absenteeism following a five-day school shutdown due to seasonal influenza in one US city found no differences between those affected and those not affected (Rodriquez et al. 2009). In Liberia, post-Ebola telephone surveys found that one month after the pandemic, one in four households reported that their children had not yet returned to school (Carvalho et al. 2020). Keith Meyers and Melissa Thomasson (2017) explored the long-term effects of the 1916 polio epidemic in the United States and found that those who were aged 14 to 17 during the epidemic had lower educational attainment in 1940. Specifically, a one standard deviation increase in the number of community cases per 10,000 people was associated with a 0.07 year decrease in educational attainment. The results, however, represent an upper-bound estimate, as effects were unable to differentiate between those who were directly afflicted with polio and those who were indirectly affected through school closures. This is due, in part, to the assumption that state-level polio morbidity (rate of disease in a population) is an accurate measure of school disruption.

Enrolment and learning can be negatively affected both by the destruction or the disruption of educational facilities. During the 2013–2016 Ebola pandemic, for example, schools in Sierra Leone were used as treatment centres, leading to hesitation among families for their children to return to school (Berry and Davis 2020). Earthquakes (Bangkok ADPC 2008) and hurricanes have also led to the destruction of schools, increasing the difficulty of resuming post-crisis education. As a result of Hurricane Mitch in 1998, 4,835 out of 20,942 public school classrooms in Honduras were destroyed (Smith 2013). In 2017, following destruction by Hurricanes Maria and Irma, Puerto Rico permanently closed approximately one quarter of its schools (Finucane et al. 2020).

## School closures and past large-scale health crises

During health crises featuring a communicable disease, school closures are a commonly used non-pharmaceutical intervention. School closures can be an effective mitigation tool to slow the transmission of an infectious disease (Jackson et al. 2013), but they are best used in combination with other strategies (Markel et al. 2007; Viner et al. 2020). Further, school closures are often initiated too late, in a reactive manner after the peak of infections have passed (Cauchemez et al. 2009). Key considerations in deciding whether to close schools include the case fatality rate^4^ and the infection rate among youth (Cauchemez et al. 2014; UK DOH 2014). The COVID-19 pandemic has seen an almost global shutdown of education systems. By mid-April 2020, 95% of countries worldwide had at least partially closed their schools (UNESCO 2020). Prior to resumption, schools in Pakistan, Indonesia, South Africa, Zambia and Malawi were expected to be closed for over 100 days (Crawfurd et al. 2020a). For South Africa, this equates with losing 25% to 57% of the “normal” school year (van der Berg and Spaull 2020). With schools closed for over 70% of the academic year, the Education Cabinet Secretary of Kenya recently declared the school year lost, with students required to repeat their classes upon reopening (Muraya 2020). In Europe, schools in France and Germany reopened after being closed for over 50 days, while in England schools were shut for just over 80 days before resuming at some capacity (Crawfurd et al. 2020a).

The breadth and length of school closures are two of many features that set the COVID-19 pandemic apart from previous large-scale health crises. In their systematic review of school closures due to seasonal or pandemic influenza, over half of the studies investigated by Charlotte Jackson and colleagues (2013) reported school closures of less than 14 days. Across eight countries during the 2009 H1N1 (“swine flu”) pandemic, school closures lasted an average of three to eight days (Cauchemez et al. 2014). In Mexico, the source country for the H1N1 pandemic, schools closed for 14 days from 27 April to 10 May (Herrera-Valdez et al. 2011). Targeted city-level closures occurred for three weeks in Bangkok, Thailand (Chieochansin et al. 2009), four days in Auckland, New Zealand (Stuart et al. 2013), and one month in Hong Kong (Wu et al. 2010). Information on school closures during the 1918–1919 influenza (“Spanish flu”) pandemic that killed at least 50 million people and infected one third of the global population (CDC n.d.-a) is difficult to find. Drawing from news articles, Alexandra Stern and colleagues (2009) found inconsistent closures across the US. While districts in Los Angeles and Denver shut down for 85 and 82 days respectively, many of the largest districts, including New York City and Chicago, decided against closing.

The regional intensity and length of closures during the 2013–2016 Ebola pandemic in West Africa make it the only education system shutdown that comes close to what the world is currently experiencing. This pandemic had more cases, deaths and recoveries than all other prior Ebola outbreaks combined (Shultz et al. 2016). Previously, Ebola had never crossed national boundaries; the 2013–2016 outbreak was the first to be recognised as a pandemic, with reported cases spread across 10 countries. Sierra Leone was the epicentre of the outbreak, with all districts in the country reporting at least one case of Ebola (Amara et al. 2017), and with 99.9% of all cases concentrated in Guinea, Liberia and Sierra Leone (Shultz et al. 2016). Schools in these three countries were closed for seven (Guinea) to nine (Sierra Leone) months, resulting in 486 to 780 lost learning hours (UNDP 2015). The education of an estimated 5 million children was influenced by the shutdowns (Rohwerder 2020).

Beyond the education system, the Ebola pandemic also took a heavy toll on other sectors of affected countries in the region. For Sierra Leone and Guinea, progress in overcoming years of turmoil and political unrest was stymied when the Ebola outbreak hit. World Bank estimates for economic growth as a percentage of gross domestic product (GDP) in 2015 were adjusted from 4.3% to –0.2% in Guinea and from 8.9% to –2.0% in Sierra Leone (Bordner 2015). In Sierra Leone, average annual household income declined from USD 336 to USD 131 over the course of the pandemic (Berry and Davis 2020). In Guinea, 850 young people lost at least one parent to Ebola (The New Humanitarian 2015) and in Sierra Leone, 5,666 lost at least one parent (Amara et al 2017). Further, 30% of children were unable to receive routine vaccines during the pandemic (CDC 2015).

## The 2013–2016 Ebola pandemic and educational enrolment in Sierra Leone and Guinea

### Study context

To explore the influence of the 2013–2016 Ebola pandemic on educational enrolment, I drew on data from Sierra Leone, Guinea and Côte d’Ivoire in West Africa. I included Côte d’Ivoire as a control country, as it borders the afflicted region but was unaffected by the pandemic. Using publicly available household survey data from directly before and after the pandemic, I applied descriptive and inferential statistics^5^ to investigate two research questions:

1. *How has the 2013–2016 Ebola pandemic influenced the enrolment patterns of school-age youth in Sierra Leone and Guinea?*
2. *Has the pandemic disproportionately affected the most marginalised youth?*

Liberia was not included in this study, as household surveys from at least two periods before and one time period following the Ebola pandemic were not available. The household surveys I drew on included both the USAID Demographic Health Surveys (DHS) Program^6^ and the UNICEF Multiple Indicator Cluster Survey (MICS).^7^ The DHS and MICS both provide nationally representative data. Conducted continuously, the two surveys have increasingly taken to aligning their questionnaires and collecting data in different years to provide a more coherent picture of country activity and trends (Lisowska 2016). In contrast to national assessments, the DHS and MICS, as household surveys, are able to capture those individuals who are not currently attending school. Prior research has combined DHS and MICS data to explore changes in educational enrolment (Putnick and Bornstein 2015; Smith-Greenaway and Heckert 2013). Additionally, the two surveys act as the primary sources of data for UNESCO’s World Inequality Database on Education (WIDE).^8^

For this study, in each survey and year, I limited the sample to the school-age population. In Sierra Leone and Côte d’Ivoire, the overall school-age population is age 6–18, with ages 6 to 11 representing primary school and ages 12 to 18 representing secondary school. The comparative numbers in Guinea are age 7–19 for overall school age, with ages 7 to 12 for primary school and ages 13 to 19 for secondary school (UIS n.d.). The multiple survey years allow a cohort-level comparative analysis. A breakdown of descriptive statistics in pandemic-affected countries is provided later in this article.

I calculated education status by disaggregating (separating) the identified age group into three categories:

1. *Currently enrolled* – those who have attended school at some time during the current school year. For example, if the current school year is 2016/2017, a currently enrolled student has attended school at least once during that year.
2. *Dropout* – those who have previously attended school but have not attended at any time during the current school year. For example, if the current school year is 2016/2017, a student who has dropped out has not attended at least one day during that year, but has attended during previous years (i.e. 2014/2015 or 2015/2016).
3. *Never enrolled* – those who have never enrolled in school.

Primary- and secondary-age enrolment in this study therefore differs from primary and secondary net enrolment, as my main concern was whether youth were attending school (I did not consider the level at which they attended).

Demographic categories for this study were drawn from past literature but limited to those that provide comparative measures over survey type and year. As a result, I chose four categories: sex, location, orphan status and wealth. *Sex* is a binary variable coded 1 for female and 0 for male. Location is a binary variable coded 1 for rural and 0 for urban. *Orphan status* was calculated from survey questions that asked whether the identified youth’s biological mother and biological father were still alive. If both biological parents were dead, the youth was considered an orphan (coded 1); if any biological parent was alive, the youth was a non-orphan (coded 0). Finally, *wealth* was measured through MICS- and DHS-provided wealth quintiles. Quintile 1 included the poorest youth, while Quintile 5 included the wealthiest youth.

## Method

To address the two research questions, I used a combination of descriptive and inferential statistics. Proportions were examined over time to provide an overview in response to Research Question 1. For Research Question 2, I calculated change in dropout rate between pre- and post-pandemic samples. Dropout rate here equals the number who have dropped out divided by those who have ever attended (dropped out + currently enrolled). To include multiple demographic variables simultaneously and disentangle the effect of the Ebola pandemic from any effect related to change over time, I ran a series of inferential analyses.

First, I used *logistic regression* to predict the association between demographic groups and the likelihood of being included in the dropout category. Logistic regression is used when the dependent variable is binary and multiple predictor or independent variables are included (Sperandei 2014). The dependent variable is presented after a logit function^9^ transforms the outcome within the possible range of odds or probabilities (Osborne 2012). To ease the interpretation of results, odds ratios are provided later in this article.

Second, to better tease out the effect of the Ebola pandemic on the most marginalised, I applied a *differences-in-differences analysis*. Differences-in-differences is increasingly popular in education policy research (Zhang 2010). It is often used in “natural” experiments^10^ to correct the estimate of the treatment effect (Murnane and Willett 2011). A basic differences-in-differences analysis includes two groups with observations over two time periods. One group (the treatment group) is exposed to the treatment during the second time period but not the first. The other group (the control group) is not exposed to the treatment group during either time period. The change between the two periods for the control group is then subtracted from the change for the treatment group (see Table 1). This process increases confidence in the estimate, because the differences-in-differences analysis provides a linear adjustment to “subtract out” the effect related to change over time, which can threaten the validity of the results (Murnane and Willett 2011). It ensures that “any variables that remain constant over time (but are unobserved) that are correlated with the selection decision and the outcomes variable will not bias the estimated effect” (Buckley and Shang 2002, p. 1). This includes unobserved, permanent differences between the treatment and control groups.

**Table 1:**
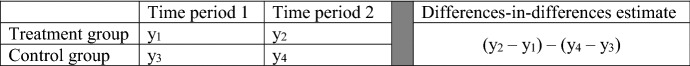
Basic differences-in-differences design

In this study the Ebola pandemic was viewed as a natural experiment, with those in outbreak-stricken countries (Guinea and Sierra Leone) identified as the exposed or treatment group. Following the work of Liang Zhang (2010) and others (Lavrijsen and Nicaise 2016; Salinas and Solé-Ollé 2018), I chose a comparable, proximate region or country as the control group. I selected Côte d’Ivoire, as it borders two of the most affected countries (Liberia and Guinea), yet did not report a single case of Ebola (CDC n.d.-b). In addition, similar to Guinea and Sierra Leone, available DHS or MICS surveys for Côte d’Ivoire were completed directly before and after the pandemic, thus covering the necessary time periods.

To capture the “within group” over time estimates, I employed a *linear probability model*. A linear probability model uses ordinary least squares regression^11^ to predict the probability of a binary outcome variable. This approach makes it easier to interpret the calculated differences and has been regularly used in differences-in-differences analysis (for examples, see Avdeev 2020 and Görlitz and Gravert 2018). There are two primary concerns with a linear probability model. First, it is possible for predicted probabilities to fall outside of the binary (0 or 1) range of the outcome variable. Second, there is inherent heteroscedasticity,^12^ which can lead to inconsistent standard error estimates (Huang 2019). In this analysis, the first concern was not as pressing, as the main estimate of interest was the differences-in-differences and not the predicted probability; the second concern was partially mitigated through calculating robust standard errors during the analysis. Finally, once the last differences-in-differences estimator is calculated, it is compared to its own standard error to calculate a t-statistic^13^ and to check for statistical significance (Murnane and Willett 2011) (See Equation 1).

**Equation** 1: Calculating t-statistic for differences-in-differences analysis

1 $$\begin{aligned} t = & {\text{differences - in - differences}}\;{\text{estimate}} \\ & \, \div \sqrt {\left[ {\left( {{\text{standard error}}_{{{\text{treatment}};{\text{time 1}}}} } \right)^{{\text{2}}} + \left( {{\text{standard error}}_{{{\text{treatment}};{\text{time 2}}}} } \right)^{{\text{2}}} + \left( {{\text{standard error}}_{{{\text{control}};{\text{time 1}}}} } \right)^{{\text{2}}} + \left( {{\text{standard error}}_{{{\text{control}};{\text{time 2}}}} } \right)^{{\text{2}}} } \right]} \\ \end{aligned}$$

There are two key assumptions underlying a differences-in-differences approach. First, it is assumed that there are no other time-varying factors affecting the outcome once change in time has been controlled by subtracting it out in the analysis. Second, it is assumed that the effect of time is “group-invariant”, suggesting that without the treatment, the same change over time is expected in both the control and treatment groups (Zhang 2010).

## Results

### The 2013–2016 Ebola pandemic and school enrolment patterns

**Fig. 1:**
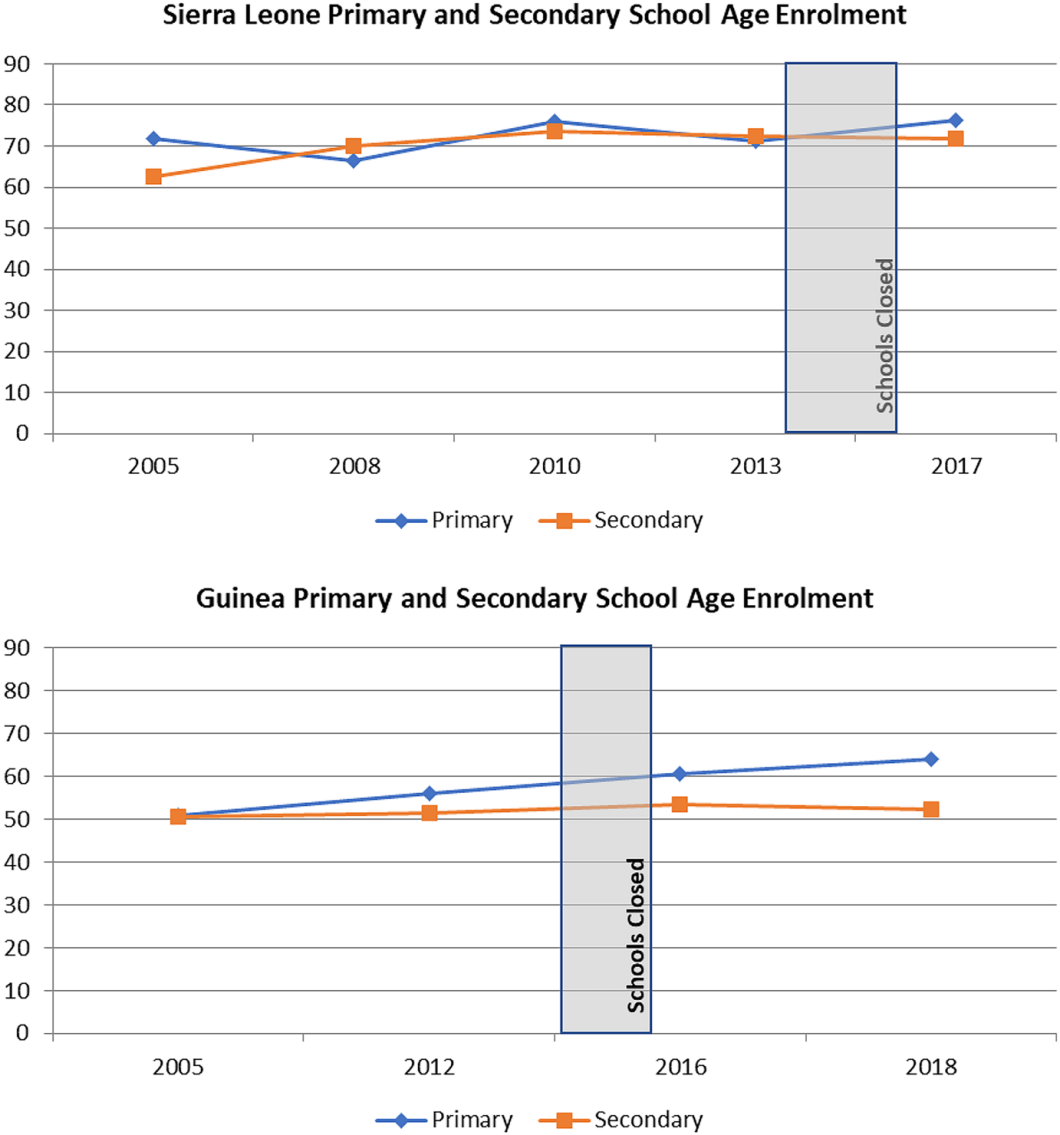
School enrolment patterns in Sierra Leone and Guinea by age group and country, pre- and post-Ebola pandemic

Figure 1 overlaps school closures during the Ebola pandemic with enrolment rates of school-age youth in Sierra Leone and Guinea between 2005 and 2018. The World Health Organization (WHO 2014a) reports that the first case of Ebola in Sierra Leone occurred in May 2014. Schools throughout the country shut down immediately thereafter in June 2014 and remained closed until March 2015 (UNDP 2015). Guinea has been identified as “ground zero” for the outbreak. We now know that a two-year-old boy from the village of Meliandou was the country’s first case, in December 2013 (Shultz et al. 2016). However, it was not until March 2014 that Guinea confirmed that the deadly fever sweeping across one of its regions had been identified as Ebola (WHO 2014b). Schools were closed in June 2014 and reopened in January 2015 (UNDP 2015). West Africa was declared Ebola-free in January 2016 (WHO 2016).

Throughout this time period (2005–2018), a larger percentage of primary and secondary-age youth were enrolled in school in Sierra Leone than Guinea. Since 2005, both primary- and secondary-age enrolment in Sierra Leone and Guinea increased, albeit slightly. This was likely driven by a reduction in school-age youth who had never enrolled in school: from 28.2% to 20.0% in Sierra Leone and from 45.5% to 33.9% in Guinea.

In examining Fig. 1, there is no clear connection between the Ebola pandemic and school enrolment in either country. When focusing on years directly before and after the pandemic, however, we can see some small differences in the patterns by school age group. For example, in both Sierra Leone and Guinea, primary-age enrolment increased after the pandemic. In contrast, secondary-age enrolment saw a slight dip in Sierra Leone, from 73.0% in 2013 to 71.8% in 2017, while in Guinea the gap between enrolment of primary- and secondary-age youth widened during the pandemic. While this macro-level aggregation of enrolment by country may hint at a few post-pandemic differences, it tells us nothing about the change in composition of school attendees.

### Dropout rate changes among marginalised groups

To examine how the Ebola pandemic affected the most marginalised, Tables 2 and 3 explore the change in dropout rates by sex, location, orphan status and wealth. Descriptive statistics included in these tables highlight trends; whether or not these trends are statistically significant is examined during the inferential analysis (starting with Table 4). Tables 2 and 3 provide information on dropout rates from the household survey directly before (2013 in Sierra Leone and 2012 in Guinea) and directly after the pandemic (2017 in Sierra Leone and 2016 in Guinea). Dropout rates are calculated as a percentage of those who have ever been to school.

**Table 2:**
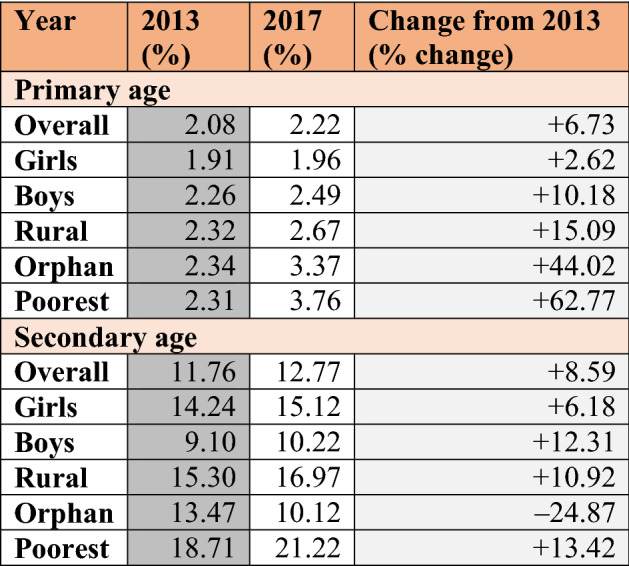
Change in dropout rate by age group: Sierra Leone

**Table 3:**
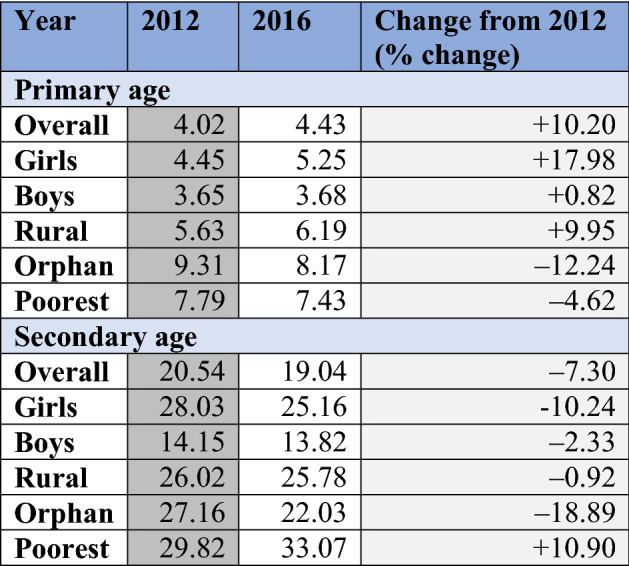
Change in dropout rate by age group: Guinea

**Table 4:**
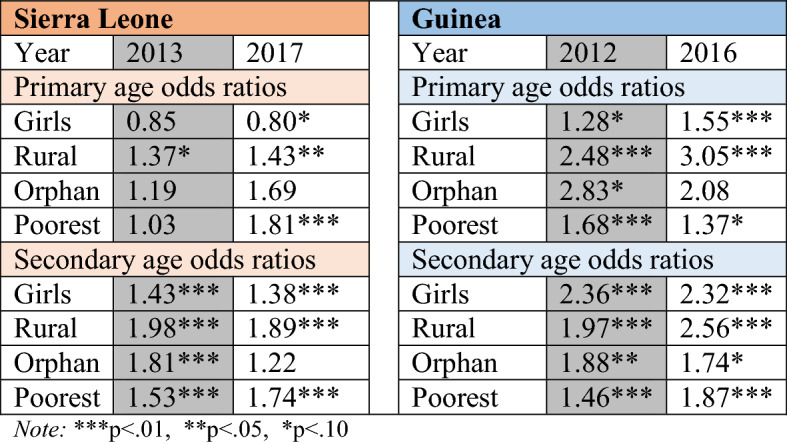
Predicting dropout by age group in Sierra Leone and Guinea

In Sierra Leone (Table 2), dropout rates for both primary- and secondary-age youth increased following the pandemic. Although the dropout rate remained relatively low among the primary age group, some subgroups saw disproportionate spikes post-pandemic. This included primary age youth in rural areas (where dropout rates increased by 15.09%), along with orphans (44.02% increase) and those in the poorest quintile (62.77% increase). As dropout rates for the secondary age group started from a higher baseline (overall dropout rate for primary age in 2013 was 2.08% compared to 11.76% for secondary age), the percentage change was not as drastic. However, similar to the primary age group, we can see that boys appear to have been more affected, as well as youth in rural areas (10.92% increase in dropout rate) and in the poorest category (13.42% increase). The change in dropout rate for orphans, and its distinction from the primary age group, may be due to the small sample size of orphans in the dataset. This makes conclusions on effects for orphans very sensitive to small shifts across surveys and suggests all results related to the orphan category should be interpreted with caution.

In Guinea (Table 3), the greatest immediate change in dropout rate occurred in primary-age students and, relative to the overall change in dropout rate for that age group (10.20% increase), only girls saw an excessive increase in dropout rate (17.98% increase). The other increase of over 10% was found among the poorest secondary-age youth. Immediately following the Ebola pandemic, the dropout rate for individuals in this category increased by 10.90% (relative to a 7.30% decrease in overall secondary dropout rate).

Taken together, the results from Tables 2 and 3 suggest that youth in the poorest households saw the largest increase in post-pandemic dropout rates. Those in rural areas were also susceptible to disruptions, while gender differences seemed to vary by country and age group. Finally, the Ebola pandemic appears to have done more harm to the dropout rate in Sierra Leone than in Guinea.

Table 4 uses logistic regression to predict who was likely to drop out pre- and post-pandemic in Sierra Leone and Guinea. As girls, youth in rural areas, orphans and those in the poorest quintile represent traditionally marginalised groups in each country, it is not surprising that most odds ratios are above 1.00 and significant, suggesting that these students were more likely than the relative reference categories to drop out. Looking at differences in odds ratios before and after the Ebola pandemic can draw attention to additional vulnerability experienced by groups. The results reinforce those found in Tables 2 and 3, suggesting that the poorest were the hardest hit. For primary-age youth in the bottom wealth quintile in Sierra Leone, the odds that they would drop out increased from 1.03 times greater than all other youth (not significantly different) to 1.81 times greater (p<.01). The likelihood that the poorest secondary-age youth would drop out in Sierra Leone also increased, from 1.54 times greater (p<.01) than the reference group to 1.75 times greater (p<.01), a trend that is also found among the poorest secondary-age youth in Guinea.

Table 4 also illustrates the precarious position of youth in rural Guinea. Among secondary-age youth in the country, the odds of those in rural areas dropping out increased from 1.46 times greater (p<.01) than urban youth pre-outbreak to 1.87 times greater (p<.01) immediately following the pandemic. For rural primary-age youth, the related odds ratio increased from 2.48 (p<.01) to 3.05 (p<.01) over the same period.

To evaluate whether the dropout changes simply reflect expected changes over the time period under consideration or can be more confidently attributed to the Ebola pandemic, I conducted a differences-in-differences analysis. Given the consistent negative relationship illustrated in Tables 2, 3 and 4, I focused this analysis on secondary-age youth from the poorest households. As mentioned earlier, the differences-in-differences analysis approached the pandemic as a natural experiment. I chose Côte d’Ivoire as the control country as it borders two of the three most affected countries (Liberia and Guinea), yet did not report a single case (CDC n.d.-b). Similar to Guinea and Sierra Leone, household survey data for Côte d’Ivoire were available from directly before (2012) and after the pandemic (2016).

**Fig. 2:**
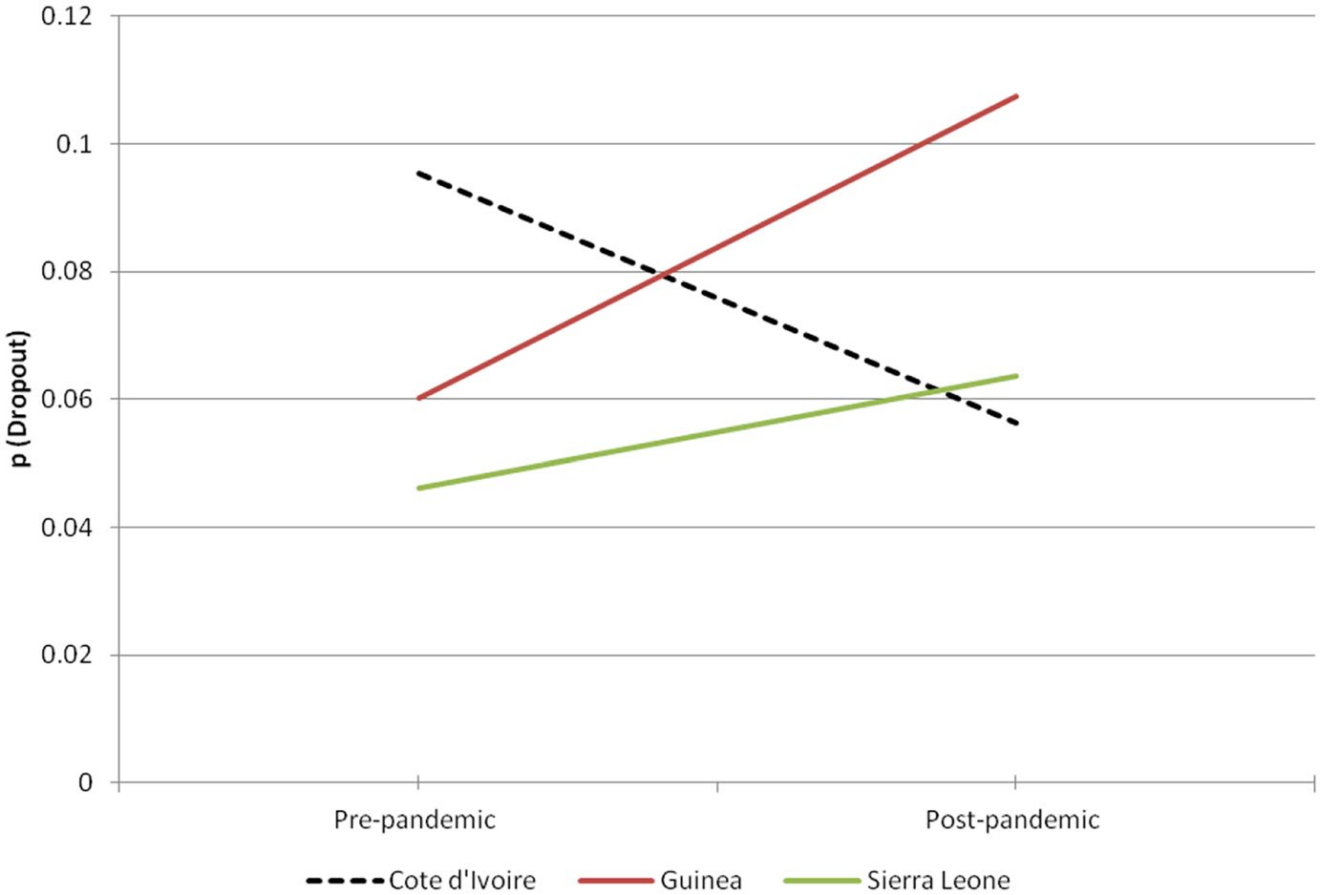
Probability of dropping out for secondary-age youth from the poorest families by country, pre- and post-pandemic. *Note:* Probability estimates control for sex, orphan status and location

Figure 2 compares the sample probability that secondary-age youth from the poorest households would drop out of school, across the two affected (treatment) countries (Guinea and Sierra Leone) and the control country (Côte d’Ivoire). Pre-outbreak, Côte d’Ivoire had the highest probability, with nearly 1 in 10 secondary-age youth from poor families predicted to drop out. Over time, the predicted probability has decreased for this group. Both Guinea and Sierra Leone stand in sharp contrast to Côte d’Ivoire. In the two countries affected by the pandemic, secondary-age youth from the poorest households were less likely to drop out pre-outbreak. However, directly following the pandemic, predicted probabilities increased, nearly doubling from 6% to 11% in Guinea.

The differences-in-differences approach removes the expected effect due to change over time from the treatment effect. This is done through taking two differences, as detailed in Table 5. Given the patterns present in Fig. 2, it is not surprising that the differences-in-differences estimate of the effect of the Ebola pandemic on the probability of poorer secondary-age youth dropping out is actually higher than the observed first-order difference. The results suggest that the pandemic was associated with an 8.6 percentage point increase in the probability of the poorest secondary-age youth in Guinea dropping out, and a 5.6 percentage point increase in Sierra Leone.

**Table 5:** Differences-in-differences estimate of the impact of the Ebola pandemic on whether secondary-age youth from the poorest households would drop out of school

Country	Pre-/post-pandemic	Affected by the pandemic?	Probability of poorest secondary-age youth dropout (standard error)	Between-group differences in probabilities	Differences-in-differences estimate (standard error)	Differences-in-differences p-value^**+**^
Guinea	Pre-pandemic	Yes	0.060 (0.025)	0.047	0.086 (0.044)	0.026**
Guinea	Post-pandemic	Yes	0.108 (0.024)			
Côte d’Ivoire	Pre-pandemic	No	0.095 (0.022)	–0.039		
Côte d’Ivoire	Post-pandemic	No	0.056 (0.016)		0.056 (0.032)	0.038**
Sierra Leone	Pre-pandemic	Yes	0.046 (0.012)	0.017		
Sierra Leone	Post-pandemic	Yes	0.064 (0.012)			

*Note*: Linear probability model controlling for sex, location and orphan status. Côte-d’Ivoire used as control country for separate differences-in-differences estimates for Guinea and Sierra Leone. ** p<.05 ^+^ one-tailed test

To examine what these numbers mean in practice, I used the pre- and post-pandemic dropout rates for both countries to estimate the net decrease in enrolment numbers among the poorest youth. In Sierra Leone, the increased dropout rates were exacerbated by the shift in overall poverty towards youth after Ebola (see Table 6). Changes in dropout rates among the poorest in Sierra Leone indicate that approximately 4,530 more primary-age youth and 5,770 more secondary-age youth in the bottom wealth quintile dropped out of school than would have been expected pre-outbreak. In Guinea, the impact of the Ebola pandemic on the poorest secondary-age youth was an additional 11,640 dropouts.

**Table 6:**
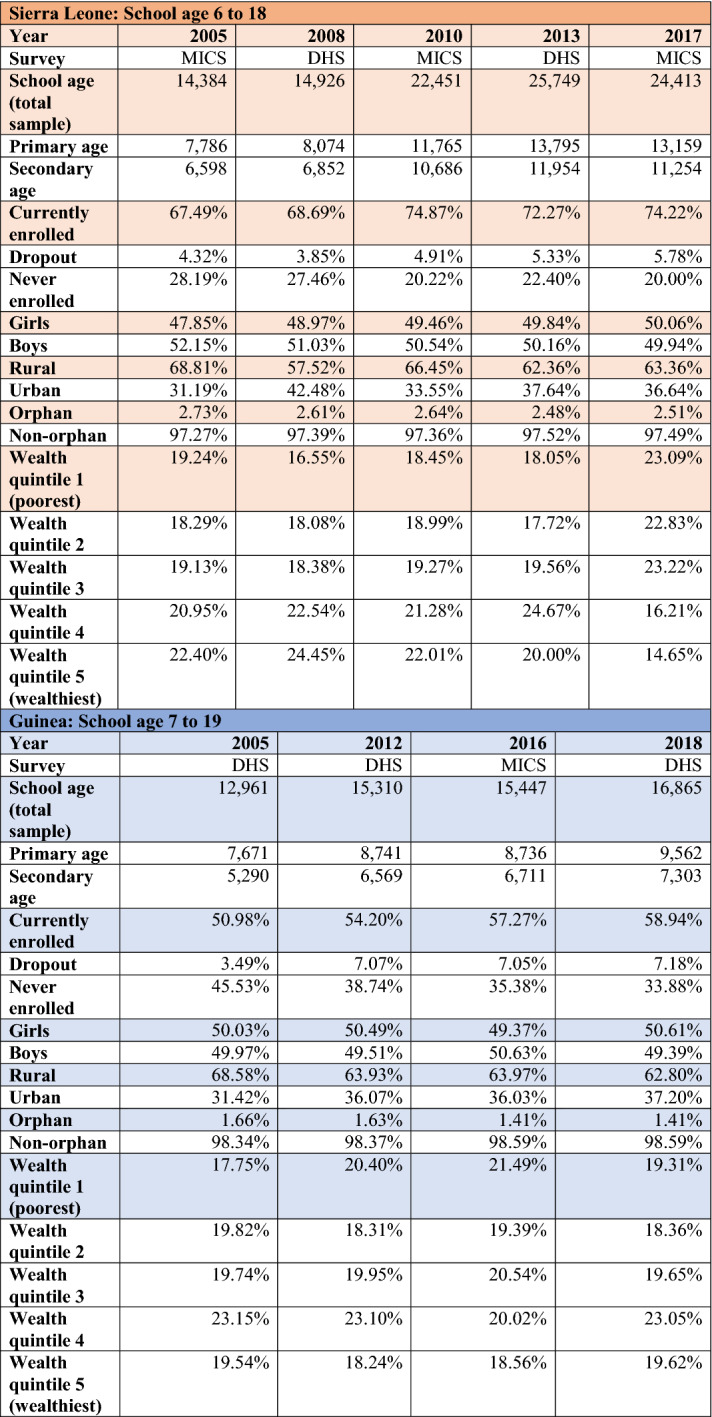
Descriptive statistics for Sierra Leone and Guinea

## Discussion and potential lessons for the COVID-19 pandemic

During the 2013–2016 Ebola pandemic in Guinea and Sierra Leone, changes in school enrolment appear to be disproportionately concentrated among secondary students in poorer families and in rural areas. Although there is no clear overall discontinuity in enrolment patterns at the national level, disaggregating and investigating proclivity to drop out indicates that marginalised groups were substantially influenced by the pandemic and associated school disruptions. In practice, this leads to expanding inequality in access for those in poor households.

The results from the study I have presented here suggest that secondary-age youth may be particularly vulnerable to school disruptions. Comparing pre- and post-pandemic estimates, the probability that secondary-age youth from the poorest families would drop out increased by 5.6 percentage points in Sierra Leone and 8.6 percentage points in Guinea. This may be due, in part, to older youth having taken on additional paid labour during the Ebola shutdowns. In Sierra Leone, many of the 3 million children in affected communities engaged in economic activity for household survival (Government of Sierra Leone 2015). Children reported increased pressure to participate in ways of supporting their families (Hallgarten 2020), and these new responsibilities may have been retained given the corresponding economic crisis many experienced. In Guinea, the impact on the most marginalised further exacerbated challenges for this vulnerable group in accessing school. Relative to Sierra Leone, the low enrolment numbers pre-outbreak in Guinea suggest that the most marginalised population have yet to access education.

Limited findings specific to orphans and girls were surprising, given the past literature suggesting that both groups were significantly negatively affected by the Ebola pandemic. For orphans, this may be due to how the group was defined. While a large number of children lost one parent to Ebola, limiting the definition of an orphan to a youth without both biological parents severely reduced the sample size. This led to more imprecise results that were prone to fluctuations. In addition, while many young people were orphaned during the crisis, some research suggests that dropout prevention programmes targeting orphans in Sierra Leone have been effective (Hallgarten 2020).

For girls, it is well documented that teen pregnancies rose in Sierra Leone during the pandemic, with estimates of new teenage pregnancies ranging from 14,000 to 20,000 (Parnebjork 2016). Directly following the resumption of education in the country, the Minister of Education forbade visibly pregnant girls from re-enrolling; this ban on attendance for pregnant teenagers was not lifted until 2020 (BBC 2020). So why have household surveys not captured this rise in school dropouts? It may be due to the level of detail in which the questions are asked. Both the DHS and the MICS ask about school attendance during the current year, but do not define what a school is. After the Minister of Education in Sierra Leone banned re-enrolment for pregnant girls, a parallel route to education through non-formal learning centres was established; 14,500 girls were enrolled (Parnebjork 2016), limiting the number of dropouts as defined in the current study. Finally, while the differences-in-differences approach allows us to more confidently claim an observed effect, it is general to the Ebola pandemic. Thus, differences in dropout rate cannot be directly attributed solely to school closures and are likely to be influenced by multiple compounding challenges brought by the pandemic. Future research should target cohorts that were in the more vulnerable category (e.g. secondary-age youth from the poorest households) when the outbreak hit and use longitudinal data to follow their post-pandemic path to more clearly illustrate long-term effects.

Before exploring what education planners preparing for school restarts during the COVID-19 pandemic can learn from the Ebola pandemic, we need to compare the two diseases. As the focus is on school-age populations, lessons are more likely to be transferable if the diseases have similar youth infection rates, case fatality rates and modes of transmission. During the Ebola pandemic, infections were concentrated in young adults. In Sierra Leone, the two most infected age groups were 25–29 year-olds and 15–19 year-olds (Amara et al. 2017). Across West Africa, 20% of Ebola cases occurred in children below the age of 15 (CDC 2015). The disease is transmitted through direct contact with infected skin, blood or other bodily fluids; this includes the shedding of infected cells left on clothing or bedding (Shultz et al. 2016). Individuals have to be symptomatic to be infectious (Berry and Davis 2020), and they remain infectious as long as the virus is present, for up to 61 days (Shultz et al. 2016). The case fatality rate for Ebola is high, making it a greatly feared disease. Across the 2013–2016 Ebola pandemic, 39.5% of those infected died; in Sierra Leone the rate was 28% and in Guinea, 66.7% (Shultz et al. 2016). Finally, survivors are immune to that strand of Ebola for at least ten years (ibid.).

What we are learning about COVID-19 is rapidly changing. At the time of writing, there is evidence to suggest that the infection rate among youth may be lower. Ten per cent of global cases have been in the 15 to 29 age group, and those below age 15 have made up less than 2% of total cases (WHO 2020a). Exploring age-specific cases across six countries, Nicholas Davies and colleagues (2020) found that those under the age of 20 were only half as susceptible to infection as those over 20. In Iceland and Japan, children with extended exposure to the virus were less likely to test positive than similarly exposed adults (Crawfurd et al. 2020b). The virus is transmitted through direct contact with infected persons and through droplets expelled when coughing, sneezing or talking (Rothan and Byrareddy 2020), allowing it to spread rapidly. Infected individuals are not always symptomatic. This is especially true in youth, where nearly 80% of infected 10–19-year-olds across 32 geographic settings were either asymptomatic or displaying symptoms at a sub-clinical level (Davies et al. 2020). Asymptomatic carriers can still transmit the virus, but the efficiency of transmission relative to those displaying symptoms is currently unclear. The global case fatality rate for COVID-19 is 5.33% as of 18 June 2020 (Worldometer 2020); however, mortality appears heavily concentrated in the older population. While the global median age for cases is 55, the median age of death is 81, with less than 2% of total deaths occurring before the age of 40 (WHO 2020a). Finally, it is unclear whether immunity, if any, is present in survivors (WHO 2020b).

Differences between Ebola and COVID-19 suggest that personal hygiene measures are essential for school reopening; however, “social” (physical) distancing has only been necessary during the COVID-19 pandemic (Berry and Davis 2020). Ebola is easier to identify and isolate. As youth with COVID-19 are disproportionately asymptomatic, there is increased uncertainty as to whether infected individuals are attending school. The fear over the significant Ebola case fatality rate may have deterred individuals from attending school, but could have also prompted action as people recognised the severity of the situation and adjusted their behaviour accordingly. In contrast, the relatively mild mortality rate in youth with COVID-19, combined with its more asymptomatic display, may lead to false confidence that individuals are beyond harm.

For education planners, this suggests that families may be more comfortable returning to school post-COVID-19 relative to the Ebola pandemic. Children are less likely to feel or look sick and, with mortality concentrated in the elderly, young people are less likely to be orphaned during the current pandemic. This could reduce the stigmatisation that kept some students in West Africa from re-enrolling post-Ebola (Hallgarten 2020). The safety and condition of facilities may also deter the return to school. If parents are not certain that school is a secure place for their children, they may decide to wait. Additionally, some schools may be in a state of disrepair following a long shutdown. This could be especially true in rural areas. Social distancing measures may also discourage students from returning, or push them out of school once they have arrived. Distance-related policies put in place after the Ebola pandemic restricted class sizes and led to increased private school enrolment, further impacting the poorest students (Santos and Novelli 2017).

Challenges for the poorest and most marginalised students have likely been compounded during the COVID-19 shutdowns. Negative indirect consequences of school closures (Berkman 2008), such as increased physical abuse and sexual exploitation (Hallgarten 2020; Roberton et al. 2020; van der Berg and Spaull 2020), and disproportionate engagement with remote learning (Giannini 2020; Herold 2020), may accelerate the exit of youth from the educational system. To mitigate the impact of the COVID-19 pandemic on those poorer students most likely to drop out, a comprehensive, long-term package of financial support is needed. Targeted approaches may include cash transfers for the most vulnerable, increased provision of school meals, and decreased or eliminated school fees (Carvalho et al. 2020). As the COVID-19 health crisis is compounded by and accelerates an economic crisis, the additional investment in education necessary to ensure those most at risk return to school may be difficult. Immediately following crises, recovery efforts tend to focus on areas such as health and sanitation over education (Hallgarten 2020). Governments will also be short on funds, forcing public sector cuts, including furloughing teachers and closing public institutions (Ibqal et al. 2020). Yet, if governments and education planners do not recognise the effects of the current pandemic on the most marginalised young people, and provide the necessary resources to ensure their return to school, the COVID-19 pandemic will certainly have long-term intergenerational impacts as inequality in access to education grows.

## Conclusion

School closures have been one of multiple societal disruptions during the COVID-19 pandemic. Reopening is likely to occur sporadically as school leaders tackle localised outbreaks and work to keep students and staff safe. Upon resumption, schools face a new reality with plans calling for reduced class sizes, staggered start times and limited interaction to permit social distancing. Closures during a large-scale health crisis are not simply a pause in learning, but an experience that is disproportionately detrimental to those on the margins. Learning gaps will widen, as *ad hoc* emergency attempts to set up remote learning during school closures have largely evaded the most marginalised and, in scanning the faces in the room, we will need to ask ourselves who is missing. The results from this study of the 2013–2016 Ebola pandemic suggest that the answer to that question is likely to be older (secondary-age) students from the poorest households. Recovery for these students is unlikely to be successful if all attention and resources are solely directed toward plans focusing on the immediate needs of schools and students. Instead, persistent efforts must be made that include comprehensive financial support packages to aid the return of the most marginalised and mitigate the impending expansion in inequality.

## Footnotes

1 Three large, global pandemics occurred in the 20th century: the “‘Spanish influenza’ in 1918, ‘Asian influenza’ in 1957, and ‘Hong Kong influenza’ in 1968. The 1918 pandemic killed an estimated 40–50 million people worldwide. That pandemic, which was exceptional, is considered one of the deadliest disease events in human history. Subsequent pandemics were much milder, with an estimated 2 million deaths in 1957 and 1 million deaths in 1968” (WHO 2007, p. 2).

2 This article was drafted in July 2020.

3 While nearly all Ebola outbreaks have come to be classified as epidemics, the 2013–2016 crisis is understood as a pandemic. Although the majority of cases were concentrated in three countries in West Africa (Guinea, Sierra Leone and Liberia), associated cases were identified across 7 other countries on 3 continents (Shultz et al. 2016). According to *The Dictionary of Epidemiology,* an *epidemic* is defined as “the occurrence in a community or region of cases of an illness, specific health-related behaviour, or other health-related events clearly in excess of normal expectancy” (Porta 2014). A *pandemic* is defined as “an epidemic occurring worldwide, or over a very wide area, crossing international boundaries and usually affecting a large number of people” (ibid.).

4 The *case fatality rate* refers to the proportion of deaths from a particular disease compared to the total number of people diagnosed with the disease.

5 *Descriptive statistics* describe the sample being studied, while *inferential statistics* are used to draw inferences or conclusions for the larger population which the sample is supposed to represent (Urdan 2010).

6 Funded by the United States Agency for International Development (USAID), the Demographic Health Surveys (DHS) Program has been collecting and disseminates nationally representative data on health and population in developing countries since 1984. The online database is at https://dhsprogram.com/.

7 Developed by the United Nations Children's Fund (UNICEF) and launched in 1995, the Multiple Indicator Cluster Survey (MICS) provides internationally comparable data on key indicators on the well-being of children and women. The online portal is at http://mics.unicef.org/.

8 An earlier version of this database was created by the United Nations Educational, Scientific and Cultural Organization (UNESCO) in 2010. In 2018, it was relaunched as the World Inequality Database on Education (WIDE). The online portal is at https://www.education-inequalities.org/.

9 The *logit function* involves calculating the national logarithm of the odds ratio (Osborne 2012).

10 A *“natural” experiment* refers to a naturally occurring event that divides the population into exposed and non-exposed groups (Craig et al. 2017).

11 *Ordinary least squares regression* is the most commonly used type of regression. It uses the smallest sum of squared deviations from a regression line to provide a prediction of Y for a given value of X (Urdan 2010).

12 *Heteroscedasticity* refers to a situation where the error variance across the identified sample differs systematically instead of randomly.

13 A *t-statistic* is commonly used for identifying whether differences between sample means are statistically significant (Urdan 2010).
